# TRANSCRIPTOMIC AGING IN THE HRS

**DOI:** 10.1093/geroni/igz038.1612

**Published:** 2019-11-08

**Authors:** Bharat Thyagarajan

**Affiliations:** Department of Laboratory Medicine and Pathology University of Minnesota; Minneapolis, Minnesota, United States

## Abstract

Since age related perturbations in gene expression profiles have been described and transcriptomic changes in specific biological pathways have been implicated in the aging process, we performed whole transcriptome sequencing on 4000 HRS participants using RNA obtained from Paxgene tubes collected during the 2016 interview. We will describe design and implementation of innovative quality control procedures to minimize technical variability in transcriptomic measurements and monitor analytical variation in large population studies such as HRS. We will also report the distribution of transcriptomic profiles according to various demographic characteristics (age, sex, racial/ethnic and socioeconomic differences) and describe the prevalence of previously reported aging related transcriptomic signatures in HRS. We will describe the associations between transcriptomic profiles and other measures of biological aging in HRS and report how changes in cell composition can affect transcriptomic profiles observed in population studies such as HRS.

